# An Upper Bound Solution for the Compression of an Orthotropic Cylinder

**DOI:** 10.3390/ma14185253

**Published:** 2021-09-13

**Authors:** Lihui Lang, Sergei Alexandrov, Yun-Che Wang

**Affiliations:** 1 School of Mechanical Engineering and Automation, Beihang University, Beijing 100191, China; lang@buaa.edu.cn (L.L.); sergei_alexandrov@spartak.ru (S.A.); 2 Department of Civil Engineering, Academy of Engineering, Peoples’ Friendship University of Russia (RUDN University), 117198 Moscow, Russia; 3 Department of Civil Engineering, National Cheng Kung University, Tainan 70101, Taiwan

**Keywords:** upsetting, orthotropy, singularity, upper bound

## Abstract

The upper bound theorem is used in conjunction with Hill’s quadratic yield criterion for determining the force required to upset a solid cylinder. The kinematically admissible velocity field accounts for the singular behavior of the real velocity field in the vicinity of the friction surface if the maximum friction law is adopted. The regime of sticking is also taken into consideration. The effect of this regime on the upper bound limit load is revealed. In particular, the kinematically admissible velocity field that includes the regime of sticking may result in a lower upper bound than that with no sticking. The boundary value problem is classified by a great number of geometric and material parameters. Therefore, a systematic parametric analysis of the effect of these parameters on the compression force is practically impossible. An advantage of the solution found is that it provides a quick estimate of this force for any given set of parameters.

## 1. Introduction

The upsetting of solid cylinders is an important metal-forming operation [1]. Moreover, this process is used as a test for evaluating flow stress and friction [2]. An experimental technique for accurately determining the strains on the cylindrical surface and the flat ends of a cylindrical compression specimen was developed and applied in [3]. Another experimental method for evaluating strain inhomogeneity was proposed in [4]. Hot upsetting tests on steel cylindrical specimens were carried out in [5,6]. The development of damage in the upsetting of cylinders was studied in [7]. A measure of barreling appearing in the cylinders’ upsetting was used to study frictional conditions and lubricant properties in [8,9,10,11,12,13].

The experimental studies above should be complemented with theoretical solutions. For this purpose, the finite element method is widely used (for example, [2,5,6,13]. However, the upper bound theorem also provides an efficient method for finding an approximate solution to these boundary value problems. It is worthy to note that the upper bound method applies to micro-forming [14] and novel metal forming processes (for example, the process for extruding curved profiles [15]). Sometimes, the upper bound method is even more efficient than the finite element method. In particular, solutions for several materials models are singular in the vicinity of maximum friction surfaces [16,17]. Finite element solutions based on standard shape functions do not converge in this case [18,19]. On the other hand, upper bound solutions incorporate this singularity with no difficulty (for example, [20,21]). Other advantages of the upper bound method over the finite element method were summarized in the recent paper [22]. A typical disadvantage of upper bound solutions is that the regime of sticking friction is ignored (for example, [23,24,25,26,27]). On the other hand, such a regime inevitably occurs over a certain portion of the friction surface in many metal forming processes.

Cylindrical orthotropy of material properties is frequently generated in the course of such processes as axisymmetric extrusion and drawing. This material property is often taken into account in structural analysis and design (for example, [28,29,30,31,32,33]). However, as follows from the review above, the analysis of cylinder’s upsetting is usually based on isotropic models. An exception is the solution provided in [34] for the upsetting of a hollow cylinder. This solution is based on Hill’s quadratic yield criterion [35]. However, it ignores the regime of sticking. Therefore, its practical value is questionable. The present paper also adopts Hill’s quadratic yield criterion. The effect of plastic anisotropy on the force required to upset a solid cylinder is demonstrated. Moreover, the effect of the regime of sticking on the upper bound limit load is revealed. In particular, the kinematically admissible velocity field that includes this regime may result in a lower upper bound than that with no sticking.

The overall motivation of this research is to demonstrate, using a simple example, that plastic anisotropy, which is a very common property of metallic materials, should not be ignored in upper bound solutions for metal forming processes.

## 2. Statement of the Problem

**Figure 1:**
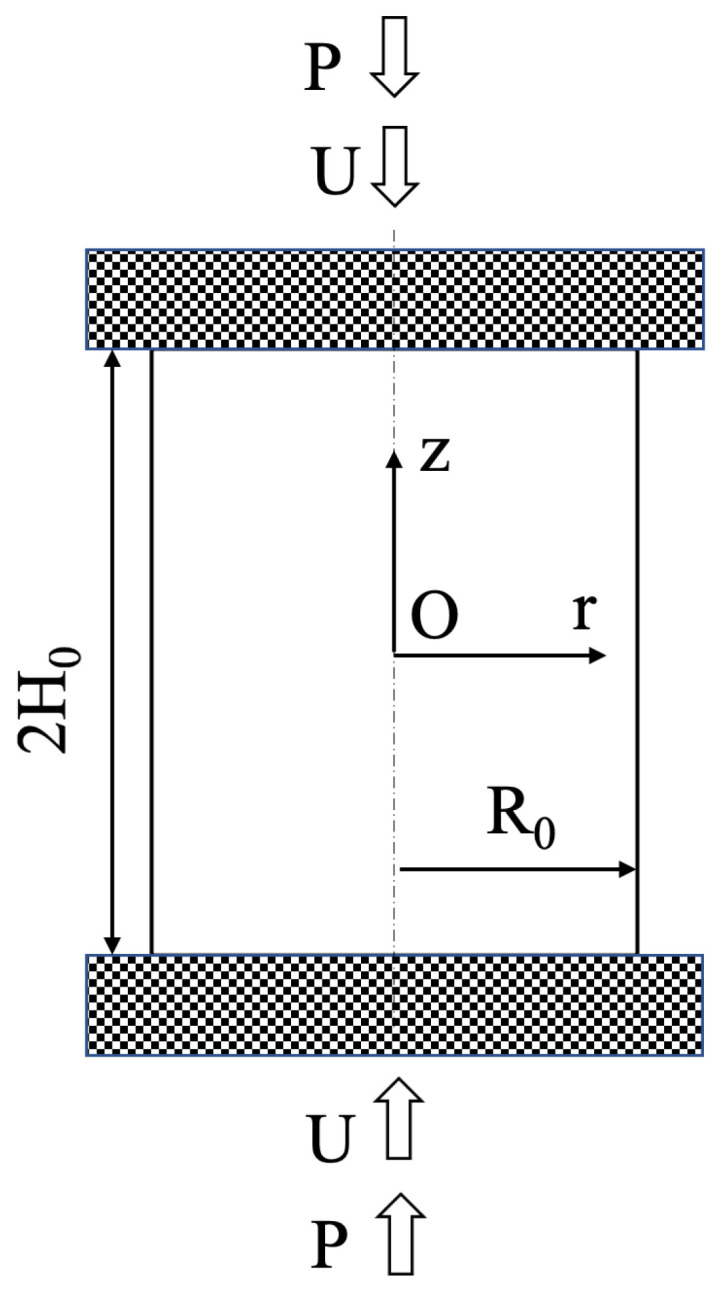
Schematic of the upsetting of a cylinder.

A circular solid cylinder is upset between two parallel rough plates (Figure 1). The radius of the cylinder is $R_{0}$, and its height is $2H_{0}$. Each plate moves with velocity *U*. The force *P* applies to each plate. This force should be found from the solution. The material of the cylinder is plastically orthotropic. The principal axes of anisotropy coincide with the radial, circumferential, and axial directions. Hill’s quadratic yield criterion is adopted [35]. Under the conditions of axial symmetry, this criterion reads as follows.

(1) $$F{\left(\sigma_{\theta \theta }-\sigma_{zz}\right)}^{2}+G{\left(\sigma_{zz}-\sigma_{rr}\right)}^{2}+H{\left(\sigma_{rr}-\sigma_{\theta \theta }\right)}^{2}+2M\sigma_{rz}^{2}=1.$$

where $\sigma_{rr}$, $\sigma_{\theta \theta }$, $\sigma_{zz}$, and $\sigma_{rz}$ are the components of the stress tensor referred to a cylindrical coordinate system (r,θ,z). The z-axis coincides with the axis of symmetry of the cylinder, and the plane z=0 coincides with the plane of symmetry of the cylinder. Since z=0 is the plane of symmetry for the flow, it is sufficient to find the solution in the region z≥0. The coefficients involved in (1) are material constants. Let Y, Θ, and *Z* be the tensile yield stresses in the radial, circumferential, and axial directions, respectively, and *S* be the shear yield stress in the rz-plane. Then, we have the following:

(2) $$2F=\frac{1}{\Theta^{2}}+\frac{1}{Z^{2}}-\frac{1}{Y^{2}},\;2G=\frac{1}{Z^{2}}+\frac{1}{Y^{2}}-\frac{1}{\Theta^{2}},\;2H=\frac{1}{Y^{2}}+\frac{1}{\Theta^{2}}-\frac{1}{Z^{2}},\;2M=\frac{1}{S^{2}}.$$

The equivalent strain rate is determined as follows [35]:

(3) $$\xi_{eq}=\sqrt{\frac{2}{3}}\sqrt{F+G+H}\sqrt{\begin{array}{c} F{\left(\frac{G\xi_{\theta \theta }-H\xi_{zz}}{FG+GH+HF}\right)}^{2}+H{\left(\frac{F\xi_{rr}-G\xi_{\theta \theta }}{FG+GH+HF}\right)}^{2}+\\ G{\left(\frac{H\xi_{zz}-F\xi_{rr}}{FG+GH+HF}\right)}^{2}+\frac{2\xi_{rz}^{2}}{M} \end{array}}.$$

where $\xi_{rr}$, $\xi_{\theta \theta }$, $\xi_{zz}$, and $\xi_{rz}$ are the components of the strain rate tensor referred to the cylindrical coordinate system. The plastic work rate per unit volume is as follows [35]:

(4) $$\omega =\sqrt{\frac{3}{2}}{\left(F+G+H\right)}^{-1/2}\xi_{eq}.$$

The velocity boundary conditions are as follows:

(5) $$u_{z}=0$$

for z=0 and

(6) $$u_{z}=-U$$

for $z=H_{0}$. Moreover, we have the following:

(7) $$u_{r}=0$$

for r=0. Here, $u_{r}$ and $u_{z}$ are the radial and axial velocities, respectively. The lateral surface of the cylinder is traction-free. Friction occurs at the surface $z=H_{0}$. It is assumed that the friction stress is equal to a constant fraction of the local shear yield stress. Taking into account the orientation of the principal axes of anisotropy, one obtains the friction stress as $\tau_{f}=mS$ where *m* is constant. Then, we have the following for $z=H_{0}$:

(8) $$\sigma_{rz}=-mS$$

The friction law (8) is valid if the regime of sliding occurs.

## 3. Kinematically Admissible Velocity Field

**Figure 2:**
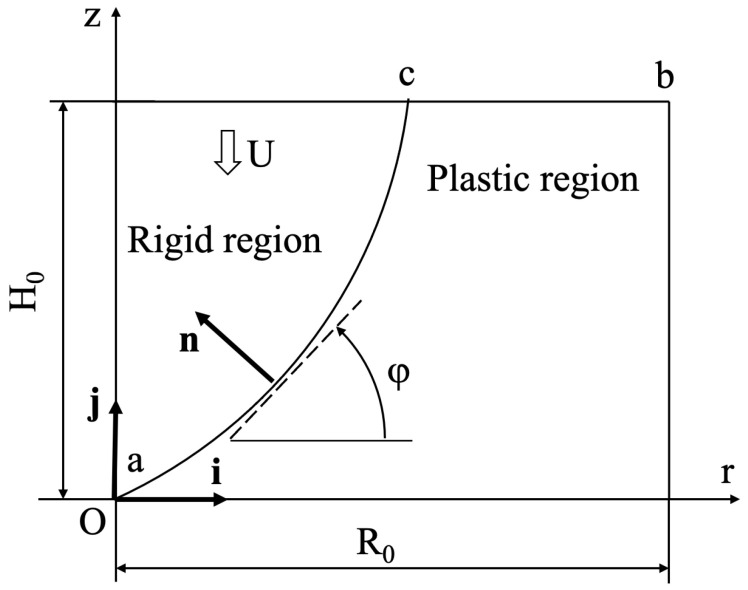
General structure of the kinematically admissible velocity field.

A general kinematically admissible velocity field for a class of axisymmetric problems was proposed in [21]. This paper deals with isotropic materials. However, the same velocity field is kinematically admissible for anisotropic materials. The general kinematically admissible velocity field can be reduced to a form appropriate for the upsetting of cylinders. The general structure of this velocity field is shown in Figure 2. The rigid region moves together with the plate. Therefore, its velocity is U. The rigid and plastic regions are separated by a velocity discontinuity line ac. This line must pass through the origin of the coordinate system. The shape of this line should be found from the solution. The solution below is valid if the radial coordinate of point c is less or equal to the radial coordinate of point b. Since the axis of symmetry belongs to the rigid region, the boundary condition (7) is satisfied. The kinematically admissible velocity field in the plastic region is as follows:

(9) $$\frac{u_{r}}{U}=\frac{\rho }{2h}+\frac{f\left(\zeta \right)}{\rho }\;\mathrm{and}\;\frac{u_{z}}{U}=-\zeta .$$

where ρ=rrR0R0, ζ=zzH0H0, h=H0H0R0R0 and f(ζ) is an arbitrary function of its argument. One can readily verify that the velocity field (9) satisfies the incompressibility equation ∂ur∂ur∂r∂r+ururrr+∂uz∂uz∂z∂z=0. In addition, the axial velocity satisfies the boundary conditions (5) and (6). The strain rate components involved in (3) are determined from (9) as follows:

(10) $$\begin{array}{c} \xi_{rr}=\frac{\partial u_{r}}{\partial r}=\frac{U}{R_{0}}\left[\frac{1}{2h}-\frac{f\left(\zeta \right)}{\rho^{2}}\right],\;\xi_{\theta \theta }=\frac{u_{r}}{r}=\frac{U}{R_{0}}\left[\frac{1}{2h}+\frac{f\left(\zeta \right)}{\rho^{2}}\right],\;\\ \xi_{zz}=\frac{\partial u_{z}}{\partial z}=-\frac{U}{H_{0}},\;\xi_{rz}=\frac{1}{2}\left(\frac{\partial u_{z}}{\partial r}+\frac{\partial u_{r}}{\partial z}\right)=\frac{U}{2H_{0}}\frac{f^{\prime }\left(\zeta \right)}{\rho }. \end{array}$$

In what follows, it is convenient to use the following dimensionless strain rate components:

(11) $${\bar{\xi }}_{rr}=\xi_{rr}\frac{H_{0}}{U},\;{\bar{\xi }}_{\theta \theta }=\xi_{\theta \theta }\frac{H_{0}}{U},\;{\bar{\xi }}_{zz}=\xi_{zz}\frac{H_{0}}{U},\;{\bar{\xi }}_{rz}=\xi_{rz}\frac{H_{0}}{U}.$$

Equations (10) and (11) combine to give the following:

(12) $${\bar{\xi }}_{rr}=\frac{1}{2}-\frac{hf\left(\zeta \right)}{\rho^{2}},\;{\bar{\xi }}_{\theta \theta }=\frac{1}{2}+\frac{hf\left(\zeta \right)}{\rho^{2}},\;{\bar{\xi }}_{zz}=-1,\;{\bar{\xi }}_{rz}=\frac{f^{\prime }\left(\zeta \right)}{2\rho }.$$

Let i and j be the unit base vectors of the axes *r* and *z*, respectively. Then, the velocity vector in the rigid region is represented as $U_{r}=-Uj$, and in the plastic region as $U_{p}=u_{r}i+u_{z}j$. The velocity normal to the velocity discontinuity line must be continuous. Then, $U_{r}\cdot n=U_{p}\cdot n$, or the following holds:

(13) $$-Uj\cdot n=u_{r}i\cdot n+u_{z}j\cdot n.$$

The velocity components $u_{r}$ and $u_{z}$ are understood to be calculated at the velocity discontinuity line using (9). From the geometry of Figure 2, we have the following:

(14) $$n=-i\sin\varphi +j\cos\varphi \;\mathrm{and}\;\tan\varphi =\frac{dz}{dr}.$$

where φ is the orientation of the tangent to the velocity discontinuity line relative to the *r*-axis. Substituting (9) and (14) into (13) and using the dimensionless coordinates yields the following:

(15) $$\frac{d\rho }{d\zeta }=\frac{h}{\left(1-\zeta \right)}\left[\frac{\rho }{2h}+\frac{f\left(\zeta \right)}{\rho }\right].$$

Using the substitution $\eta =\rho^{2}$, one transforms (15) into the following linear ordinary differential equation:

(16) $$\frac{d\eta }{d\zeta }=\frac{h}{\left(1-\zeta \right)}\left[\frac{\eta }{h}+2f\left(\zeta \right)\right].$$

The solution of this equation supplies the shape of the velocity discontinuity line. Since this line must pass through the origin of the coordinate system, the boundary condition to Equation (16) is as follows:

(17) η=0

for ζ=0. The general solution of (16) can be represented as follows:

(18) $$\eta =\eta_{ac}\left(\zeta \right)=\frac{2h\Phi \left(\zeta \right)+C}{1-\zeta }$$

where C is constant and

(19) $$\Phi \left(\zeta \right)=\int_{1}^{\zeta }f\left(\lambda \right)d\lambda .$$

The denominator in (18) approaches zero as ζ→1. Therefore, the velocity discontinuity line can reach the friction surface ζ=1 only if C=0. Then, Equation (18) becomes the following:

(20) $$\eta =\eta_{ac}\left(\zeta \right)=\frac{2h\Phi \left(\zeta \right)}{1-\zeta }.$$

This equation determines the velocity discontinuity line. Applying l’Hospital’s rule and taking into account (19), one obtains the following:

(21) $$\eta_{c}=-2hf\left(1\right).$$

where $\eta_{c}$ is the value of η at point *c* (Figure 2).

Having found the velocity discontinuity line, one can readily determine an infinitesimal length element of this line from (19) and (20) as follows:

(22) $$dL=H_{0}\sqrt{{\left[\frac{1}{2h}+\frac{f\left(\zeta \right)}{\eta_{ac}\left(\zeta \right)}\right]}^{2}\frac{\eta_{ac}\left(\zeta \right)}{{\left(1-\zeta \right)}^{2}}+1}.$$

The amount of velocity jump across the velocity discontinuity line is $\left[u\right]=\left|U_{r}-U_{p}\right|$. The velocity vectors are understood to be calculated at this line. Then, using (9) and (20), one arrives at the following:

(23) $$\left[u\right]=U\sqrt{{\left[\frac{1}{2h}+\frac{f\left(\zeta \right)}{\eta_{ac}\left(\zeta \right)}\right]}^{2}\eta_{ac}\left(\zeta \right)+{\left(1-\zeta \right)}^{2}}.$$

## 4. Upper Bound Solution

It follows from the upper bound theorem that [35]

(24) $$PU\leq W_{V}+W_{d}+W_{f}.$$

where $W_{V}$ is the plastic work rate in the plastic region, $W_{d}$ is the plastic work rate at the velocity discontinuity line, and $W_{f}$ is the plastic work rate at the friction surface. The infinitesimal volume element in the cylindrical coordinate system is dV=rdrdθdz. Using the dimensionless coordinates, one arrives at the following:

(25) $$dV=R_{0}^{2}H_{0}\rho d\rho d\theta d\zeta =\frac{R_{0}^{2}H_{0}}{2}d\eta d\theta d\zeta .$$

The plastic work rate in the plastic region is determined as follows:

(26) $$W_{V}=\;\int \int \int \;\omega dV=\pi R_{0}^{2}H_{0}\int_{0}^{1}\int_{\eta_{ac}\left(\zeta \right)}^{1}\omega d\zeta d\eta .$$

Substituting (3) into (4) and using (11), one obtains the following:

(27) $$\omega =\frac{UZ}{H_{0}}\bar{\omega }$$

where

(28) $$\bar{\omega }=\sqrt{F+G}\sqrt{\begin{array}{c} F{\left(\frac{G{\bar{\xi }}_{\theta \theta }-H{\bar{\xi }}_{zz}}{FG+GH+HF}\right)}^{2}+H{\left(\frac{F{\bar{\xi }}_{rr}-G{\bar{\xi }}_{\theta \theta }}{FG+GH+HF}\right)}^{2}+\\ G{\left(\frac{H{\bar{\xi }}_{zz}-F{\bar{\xi }}_{rr}}{FG+GH+HF}\right)}^{2}+\frac{2{\bar{\xi }}_{rz}^{2}}{M} \end{array}}.$$

where (2) is used. In (12), one can express ρ in terms of η. As a result, we have the following:

(29) $${\bar{\xi }}_{rr}=\frac{1}{2}-\frac{hf\left(\zeta \right)}{\eta },\;{\bar{\xi }}_{\theta \theta }=\frac{1}{2}+\frac{hf\left(\zeta \right)}{\eta },\;{\bar{\xi }}_{zz}=-1,\;{\bar{\xi }}_{rz}=\frac{f^{\prime }\left(\zeta \right)}{2\sqrt{\eta }}.$$

Eliminating ${\bar{\xi }}_{rr}$, ${\bar{\xi }}_{\theta \theta }$, ${\bar{\xi }}_{zz}$, and ${\bar{\xi }}_{rz}$ in (28) using (29), one arrives at $\bar{\omega }$ as a function of η and ζ. Equation (26) becomes the following:

(30) $$\frac{W_{V}}{\pi ZR_{0}^{2}U}=\int_{0}^{1}\int_{\eta_{ac}\left(\zeta \right)}^{1}\bar{\omega }d\zeta d\eta .$$

The integral here can be evaluated numerically.

The general expression for the plastic work rate at the velocity discontinuity line is as follows:

(31) $$W_{d}=2\pi \int \tau_{s}\left[u\right]rdL=2\pi R_{0}\int \tau_{s}\left[u\right]\rho dL=2\pi R_{0}\int \tau_{s}\left[u\right]\sqrt{\eta_{ac}\left(\zeta \right)}dL.$$

where $\tau_{s}$ is the shear stress on the velocity discontinuity line. It is known that [35]

(32) $$\tau_{s}=S\sqrt{1-c\sin^{2}2\varphi }$$

where

(33) $$c=1-\frac{M\left(F+H\right)}{2\left(FG+GH+HF\right)}.$$

Using some trigonometry, one transforms (32) to the following:

(34) $$\tau_{s}=S\sqrt{1-\frac{4c\cot^{2}\varphi }{{\left(1+\cot^{2}\varphi \right)}^{2}}}.$$

where the angle φ is understood to be calculated at the velocity discontinuity line. Therefore, from(14) and (16), we have the following:

(35) $$\cot\varphi =\frac{dr}{dz}=\frac{R_{0}d\rho }{H_{0}d\zeta }=\frac{1}{2h\sqrt{\eta }}\frac{d\eta }{d\zeta }=\frac{1}{\left(1-\zeta \right)}\left[\frac{\sqrt{\eta_{ac}\left(\zeta \right)}}{2h}+\frac{f\left(\zeta \right)}{\sqrt{\eta_{ac}\left(\zeta \right)}}\right].$$

Equations (22) and (23) yield the following:

(36) $$\left[u\right]dL=UH_{0}q\left(\zeta \right)d\zeta$$

where

(37) $$q\left(\zeta \right)=\left(1-\zeta \right)\left\{{\left[\frac{1}{2h}+\frac{f\left(\zeta \right)}{\eta_{ac}\left(\zeta \right)}\right]}^{2}\frac{\eta_{ac}\left(\zeta \right)}{{\left(1-\zeta \right)}^{2}}+1\right\}$$

Substituting (34) and (36) into (31) gives the following:

(38) $$\frac{W_{d}}{\pi ZR_{0}^{2}U}=\frac{2hS}{Z}\int_{0}^{1}\sqrt{1-\frac{4c\cot^{2}\varphi }{{\left(1+\cot^{2}\varphi \right)}^{2}}}q\left(\zeta \right)\sqrt{\eta_{ac}\left(\zeta \right)}d\zeta .$$

Taking into account (20), (36), and (37), one can evaluate the integral in (38) numerically.

Using (8), one can represent the plastic work rate at the friction surface as follows:

(39) $$W_{f}=2\pi mS\int u_{r}rdr=2\pi mSR_{0}^{2}\int u_{r}\rho d\rho =\pi mSR_{0}^{2}\int u_{r}d\eta .$$

The radial velocity is understood to be calculated at the friction surface. Therefore, using (9), one transforms (39) to the following:

(40) $$W_{f}=\frac{\pi mUSR_{0}^{2}}{2h}\int_{\eta_{c}}^{1}\left(\sqrt{\eta }-\frac{\eta_{c}}{\sqrt{\eta }}\right)d\eta .$$

where Equation (21) is used. The integral in (40) can be immediately evaluated to give the following:

(41) $$\frac{W_{f}}{\pi ZR_{0}^{2}U}=\frac{mS}{hZ}\left(\frac{1}{3}+\frac{2}{3}\eta_{c}^{3/2}-\eta_{c}\right).$$

Substituting (30), (38), and (41) into (24) results in the following:

(42) $$\begin{array}{c} p_{u}=\frac{P_{u}}{\pi ZR_{0}^{2}}=\int_{0}^{1}\int_{\eta_{ac}\left(\zeta \right)}^{1}\bar{\omega }d\zeta d\eta +\frac{2hS}{Z}\int_{0}^{1}\sqrt{1-\frac{4c\cot^{2}\varphi }{{\left(1+\cot^{2}\varphi \right)}^{2}}}q\left(\zeta \right)\sqrt{\eta_{ac}\left(\zeta \right)}d\zeta +\\ \frac{mS}{hZ}\left(\frac{1}{3}+\frac{2}{3}\eta_{c}^{3/2}-\eta_{c}\right). \end{array}$$

where $P_{u}$ is the upper bound of the force required to deform the cylinder, and $p_{u}$ is its dimensionless representation. The right-hand side of this equation can be found, using the procedure above for the arbitrary function f(ζ) and any set of parameters. The function f(ζ) may involve additional parameters. In this case, the right-hand side of (42) should be minimized with respect to these parameters to find the best upper bound based on the kinematically admissible velocity field chosen.

The solution above is valid if the following holds:

(43) $$0\leq \eta_{c}\leq 1.$$

The solution with no rigid region can be obtained from the solution above, with the following:

(44) $$f\left(\zeta \right)=0.$$

In this case, there is no velocity discontinuity line, and Equation (24) becomes as follows:

(45) $$P_{u}U=W_{V}+W_{f}.$$

The regime of sliding occurs over the entire friction surface. Therefore, Equations (9), (39) and (44) combine to give the following:

(46) $$W_{f}=\pi mSR_{0}^{2}\int_{0}^{1}u_{r}d\eta =\frac{\pi mSR_{0}^{2}U}{2h}\int_{0}^{1}\sqrt{\eta }d\eta =\frac{\pi mSR_{0}^{2}U}{3h}.$$

Moreover, using (27), one transforms Equation (26) to the following:

(47) $$W_{V}=\pi R_{0}^{2}UZ\int_{0}^{1}\int_{0}^{1}\bar{\omega }d\zeta d\eta .$$

Substituting (44) into (29) leads to the following:

(48) $${\bar{\xi }}_{rr}=\frac{1}{2},\;{\bar{\xi }}_{\theta \theta }=\frac{1}{2},\;{\bar{\xi }}_{zz}=-1,\;{\bar{\xi }}_{rz}=0.$$

Then, Equation (28) becomes the following:

(49) $$\bar{\omega }=\frac{\sqrt{F+G}}{\left(FG+GH+HF\right)}\sqrt{F{\left(\frac{G}{2}+H\right)}^{2}+\frac{H}{4}{\left(F-G\right)}^{2}+G{\left(H+\frac{F}{2}\right)}^{2}}.$$

Substituting (49) into (47) and integrating gives the following:

(50) $$\frac{W_{V}}{\pi R_{0}^{2}UZ}=\frac{\sqrt{F+G}}{\left(FG+GH+HF\right)}\sqrt{F{\left(\frac{G}{2}+H\right)}^{2}+\frac{H}{4}{\left(F-G\right)}^{2}+G{\left(H+\frac{F}{2}\right)}^{2}}.$$

Equations (45), (46) and (50) supply the following dimensionless upper bound limit load:

(51) $$p_{u}=\frac{\sqrt{F+G}}{\left(FG+GH+HF\right)}\sqrt{F{\left(\frac{G}{2}+H\right)}^{2}+\frac{H}{4}{\left(F-G\right)}^{2}+G{\left(H+\frac{F}{2}\right)}^{2}}+\frac{mS}{3hZ}.$$

## 5. Numerical Examples

One should choose the function f(ζ) to evaluate the right-hand side of (42). This function specifies the kinematically admissible velocity field. In general, it is advantageous that kinematically admissible velocity fields exhibit some mathematical properties of the real velocity field. The process under consideration is symmetric relative to the plane ζ=0. Therefore, the real velocity field is described by an even function of ζ. Moreover, the real velocity field is singular near the friction surface if m=1 in (8). In particular, we have the following [17]:

(52) $$u_{r}=O\left(\sqrt{1-\zeta }\right)+A$$

as ζ→1. Here, *A* is independent of ζ. The general asymptotic analysis carried out in [17] is for plane strain deformation. However, a particular solution presented in [36] shows that (44) is also valid for axisymmetric problems. One of the simplest functions satisfying the symmetry condition and (44) is the following:

(53) $$f\left(\zeta \right)=\beta_{0}-\beta_{1}\sqrt{1-\zeta^{2}}.$$

Taking into account (21), one rewrites this equation as the following:

(54) $$f\left(\zeta \right)=-\frac{\eta_{c}}{2h}-\beta_{1}\sqrt{1-\zeta^{2}}.$$

Equations (19) and (54) combine to give the following:

(55) $$\Phi \left(\zeta \right)=\frac{\eta_{c}\left(1-\zeta \right)-\beta_{1}h\zeta \sqrt{1-\zeta^{2}}+\beta_{1}h\arccos\zeta }{2h}.$$

It follows from (17) and (20) that

(56) Φ(0)=0.

Then, Equations (55) and (56) combine to give β1=−2ηc2ηcπhπh. Eliminating $\beta_{1}$ in Equations (54) and (55), one obtains the following:

(57) $$f\left(\zeta \right)=-\frac{\eta_{c}}{2h}\left(1-\frac{4}{\pi }\sqrt{1-\zeta^{2}}\right)\;\mathrm{and}\;\Phi \left(\zeta \right)=\frac{\eta_{c}}{2h}\left[1-\zeta +\frac{2}{\pi }\left(\zeta \sqrt{1-\zeta^{2}}-\arccos\zeta \right)\right].$$

Moreover,

(58) $$f^{\prime }\left(\zeta \right)=-\frac{2\eta_{c}\zeta }{\pi h\sqrt{1-\zeta^{2}}}.$$

Substituting (49) and (50) into (42), one arrives at the right-hand side of this equation as a function of ζ. Moreover, this function involves one free parameter, $\eta_{c}$. One should minimize the right-hand side of (42), taking into account (43).

The boundary value problem is classified by five dimensionless parameters: Y/Z, Θ/Z, S/Z, *h*, and *m*. Therefore, its systematic parametric analysis is practically impossible. The numerical example below includes the isotropic case, the anisotropic properties determined experimentally and reported in [35], and three sets of arbitrarily chosen yield stresses. The isotropic solution is used for showing the effect of plastic anisotropy on the limit load. The solutions for the chosen sets of yield stresses allow one to gain insight into the possible effect of plastic anisotropy on the interpretation of the corresponding friction test.

The experimental results from [37] are summarized in Table 1. Table 2 shows the chosen sets of yield stresses.

The numerical integration has used 20 Gaussian integration points and weights for the integrals involved in (30) and (38). With a given set of the parameters that classify the boundary value problem, the minimization process to find the optimal value of $\eta_{c}$ is conducted by using hundreds to thousands of $\eta_{c}$-data points in the range between 0 and 1 to calculate $p_{u}$. The number of data points determines the resolution and accuracy of the optimal value of $\eta_{c}$. Once the $\eta_{c},p_{u}$ data list is calculated, it is straightforward to find the minimum value of $p_{u}$. This value is denoted as $p_{u}^{\left(1\right)}$. The limit load found from (51) is denoted as $p_{u}^{\left(2\right)}$. Then, the solution to the boundary value problem is as follows:

(59) $$p_{u}=\min\left\{p_{u}^{\left(1\right)},p_{u}^{\left(2\right)}\right\}$$

The optimal value of $\eta_{c}$ is also of interest because it may affect the interpretation of the friction test.

**Figure 3:**
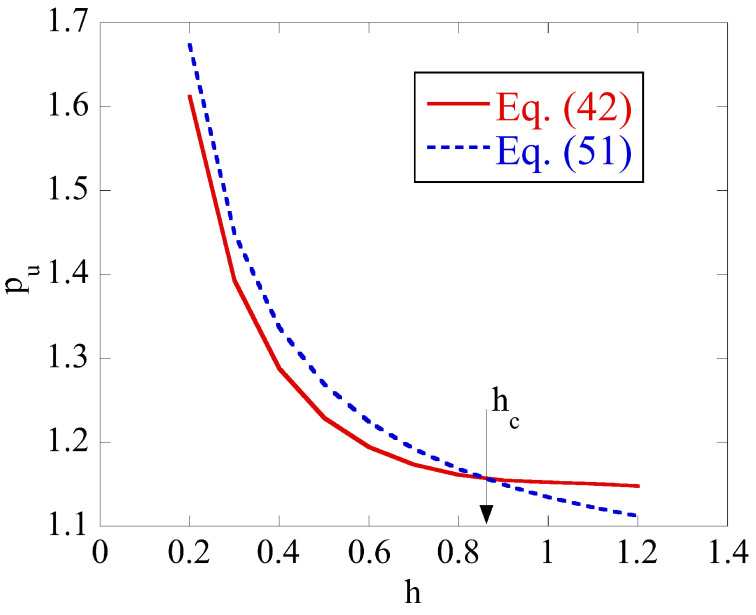
Existence of a critical value of *h* at which solutions (42) and (51) provide the same limit load for the isotropic material at m=0.7.

**Figure 4:**
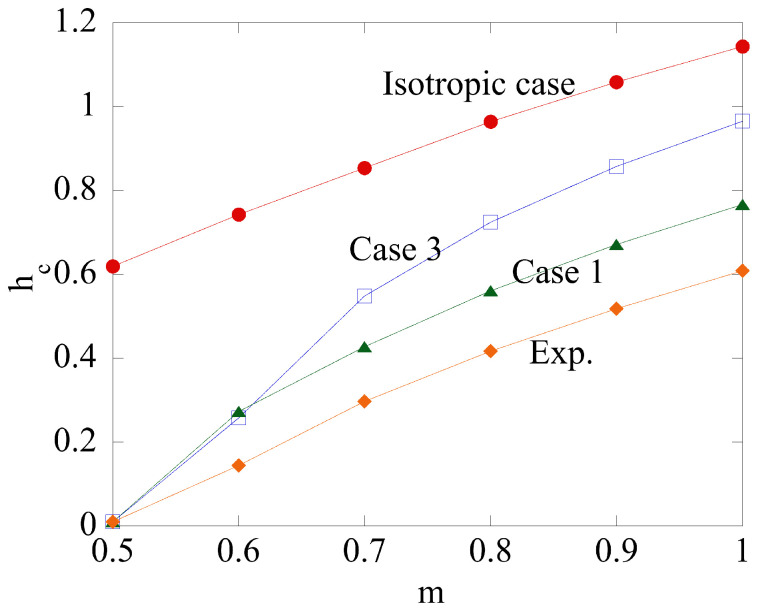
Effect of plastic anisotropy on the dependence of $h_{c}$ on *m*. No $h_{c}$ is found for Case 2 since $p_{u}^{\left(1\right)}<p_{u}^{\left(2\right)}$ in the *m* range.

It is natural to expect that $p_{u}^{\left(1\right)}=p_{u}^{\left(2\right)}$ for certain combinations of the parameters that classify the boundary value problem. Figure 3 illustrates this feature of the solution for the isotropic material at m=0.7. In this case, the only parameter that varies is *h*. It is seen from Figure 3 that $p_{u}^{\left(1\right)}=p_{u}^{\left(2\right)}$ at $h=h_{c}$. It follows from (59) that the solution (42) is valid in the range $h\leq h_{c}$ and the solution (51) in the range $h\geq h_{c}$. It is found that Case 2 results in $p_{u}^{\left(1\right)}<p_{u}^{\left(2\right)}$ if 0≤m≤1. Therefore, in this case $p_{u}=p_{u}^{\left(1\right)}$. Figure 4 depicts the dependence of $h_{c}$ on *m* for the isotropic material and three anisotropic materials whose properties are given in Table 1 and Table 2. It is seen from this figure that the range of validity of the solution with no rigid region decreases as the friction factor increases for all the cases considered.

**Figure 5:**
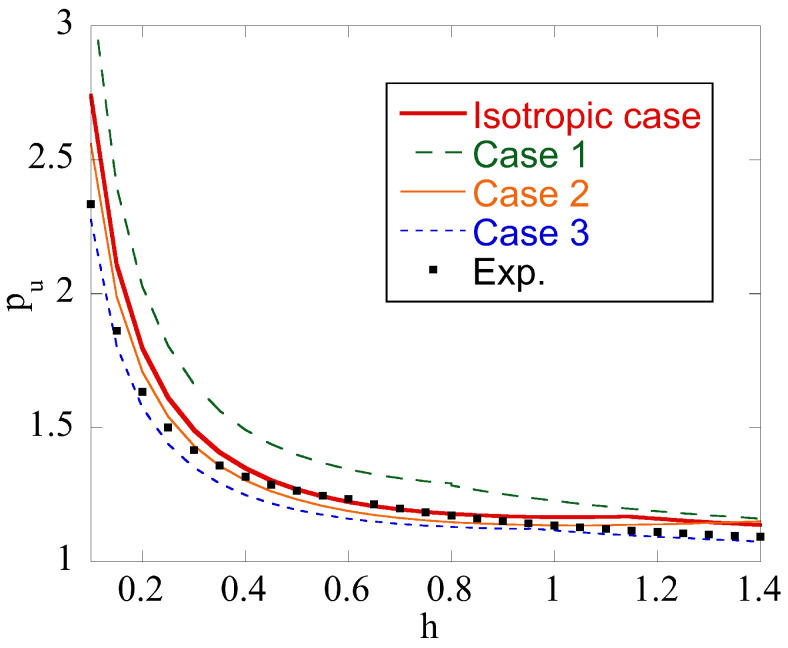
Effect of plastic anisotropy on the dimensionless limit load at m=1.

Figure 5 illustrates the limit load solution at m=1. The curves show the dependence of $p_{u}$ on *h* for the isotropic material and four anisotropic materials whose properties are given in Table 1 and Table 2. As expected, $p_{u}$ increases with *h*. The limit load for anisotropic materials may be lower or higher than that for the isotropic material. The difference between the limit load for the isotropic material and that for the anisotropic material whose properties are determined experimentally (Table 1) is between 15% and 20% in the range of *h* considered.

**Figure 6:**
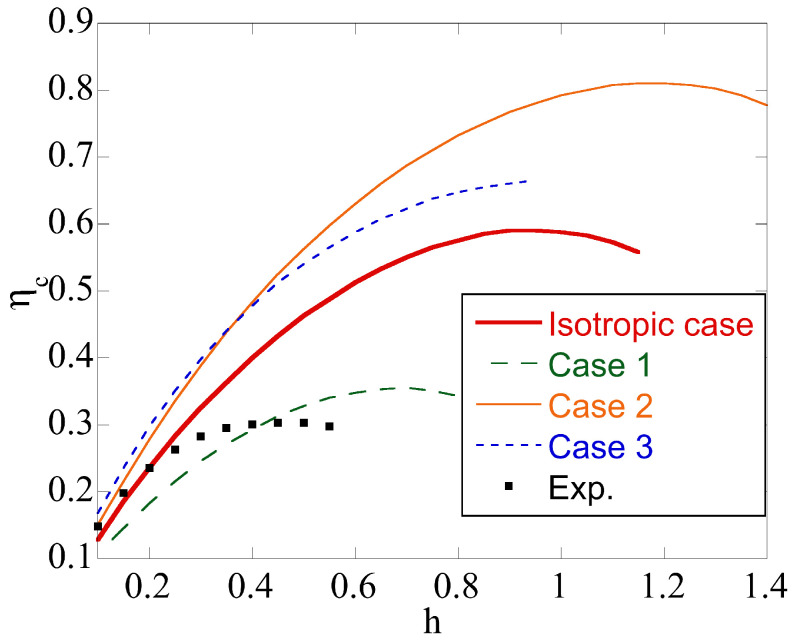
Effect of plastic anisotropy on $\eta_{c}$ at m=1.

Figure 6 depicts the variation of $\eta_{c}$ with *h* at m=1 for the isotropic material and four anisotropic materials whose properties are given in Table 1 and Table 2. It is seen from Figure 2 and Figure 6 that plastic anisotropy significantly affects the region of the friction surface where Equation (8) is valid. This feature of the model may influence the interpretation of the friction test.

## 6. Conclusions

A new upper bound solution for the upsetting of a circular cylinder is proposed. A distinguishing feature of the solution is that plastic anisotropy and the existence of a rigid region are taken into account. The existence of the rigid region automatically means that there is a region of sticking friction. From this work, the following conclusions can be drawn:

1. Plastic anisotropy affects the limit load required to deform the specimen. It may increase or decrease the limit load as compared to the isotropic case (Figure 5).
2. The upsetting of a cylinder is often used as a friction test. Plastic anisotropy significantly affects the region of sticking friction (Figure 6). Since Equation (8) is not valid in this region, this effect of plastic anisotropy should be taken into account in the interpretation of the friction test results.
3. Five dimensionless parameters classify the boundary value problem. Therefore, its systematic parametric analysis is invisible. An advantage of the proposed solution is that it quickly estimates the upper bound limit load for a given set of parameters.
4. The real velocity field is singular near the friction surface if m=1 in Equation (8). The solution proposed accounts for this singularity, which is impossible when using ordinary finite element solutions.

## Figures and Tables

**Table 1:** Experimental data from Ref. [37].

	*FZ^2^*	*HZ^2^*	*GZ^2^*	*MZ^2^*
Exp. Value	0.378	0.1	0.623	2.558

**Table 2:** Yield stresses chosen to illustrate the effect of plastic anisotropy on the interpretation of the friction test.

	Y/*Z*	Θ/*Z*	*S/Z*
Isotropic Case	1	1	$1/\sqrt{3}$
Case 1	1.2	1.5	$1.2/\sqrt{3}$
Case 2	0.8	0.7	$0.9/\sqrt{3}$
Case 3	0.8	0.9	$0.75/\sqrt{3}$

## Data Availability

The data that support the findings of this study are available from the corresponding author upon reasonable request.

## Nomenclature

i and j	unit base vectors
*m*	friction factor
n	unit vector normal to the velocity discontinuity line
$p_{u}$	dimensionless upper bound limit load
(t,θ,z)	cylindrical coordinate system
$u_{r}$ and $u_{z}$	radial and axial velocities
*F*, *G*, *H* and *M*	material parameters introduced in Equation (2)
$H_{0}$	half-height of the cylinder
*P*	force
$R_{0}$	radius of the cylinder
*S*	shear yield stress in the rz-plane
*U*	velocity of the plate
$W_{d}$	plastic work rate at the velocity discontinuity line
$W_{f}$	plastic work rate at the friction surface
$W_{v}$	plastic work rate in the plastic region
Y, Θ and *Z*	tensile yield stresses in the radial, circumferential, and axial directions
$\xi_{eq}$	equivalent strain rate
$\xi_{rr}$, $\xi_{\theta \theta }$, $\xi_{zz}$ and $\xi_{rz}$	strain rate components referred to the cylindrical coordinate system
${\bar{\xi }}_{rr}$, ${\bar{\xi }}_{\theta \theta }$, ${\bar{\xi }}_{zz}$ and ${\bar{\xi }}_{rz}$	dimensionless strain rate components introduced in Equation (11)
ρ, ζ and *h*	dimensionless quantities introduced after Equation (9)
$\sigma_{rr}$, $\sigma_{\theta \theta }$, $\sigma_{zz}$ and $\sigma_{rz}$	stress components referred to the cylindrical coordinate system
$\tau_{f}$	friction stress
φ	orientation of the tangent to the velocity discontinuity line
ω	plastic work rate per unit volume
